# Coordination of retrotransposons and type I interferon with distinct interferon pathways in dermatomyositis, systemic lupus erythematosus and autoimmune blistering disease

**DOI:** 10.1038/s41598-021-02522-6

**Published:** 2021-11-30

**Authors:** Yuko Kuriyama, Akira Shimizu, Saki Kanai, Daisuke Oikawa, Sei-ichiro Motegi, Fuminori Tokunaga, Osamu Ishikawa

**Affiliations:** 1 Department of Dermatology, Gunma University Graduate School of Medicine, Maebashi, Gunma Japan; 2 Department of Dermatology, Kanazawa Medical University, 1-1 Daigaku, Uchinada, Kahoku-gun, Ishikawa 920-0293 Japan; 3 Department of Pathobiochemistry, Graduate School of Medicine, Osaka City University, Osaka, Japan

**Keywords:** Rheumatic diseases, Skin diseases

## Abstract

Type I interferon (IFN) plays a crucial role in innate and adaptive immunity, and aberrant IFN responses are involved in systemic autoimmune diseases, such as systemic lupus erythematosus (SLE) and dermatomyositis (DM). Type I IFNs can be induced by transcribed retrotransposons. The regulation of retrotransposons and type I IFN and the downstream IFN pathways in SLE, DM, and autoimmune blistering disease (AIBD) were investigated. The gene expression levels of retrotransposons, including *LINE-1*, type I-III IFNs, and IFN-stimulated genes (ISGs) in peripheral blood cells from patients with DM (n = 24), SLE (n = 19), AIBD (n = 14) and healthy controls (HCs, n = 10) were assessed by quantitative polymerase chain reaction. Upregulation of retrotransposons and IFNs was detected in DM patient samples, as is characteristic, compared to HCs; however, ISGs were not uniformly upregulated. In contrast, retrotransposons and IFNs, except for type II IFN, such as IFN-γ, were not upregulated in SLE. In AIBD, only some retrotransposons and type I interferons were upregulated. The DM, SLE, and AIBD samples showed coordinated expression of retrotransposons and type I IFNs and distinct spectra of IFN signaling. A positive correlation between LINE-1 and IFN-β1 was also detected in human cell lines. These factors may participate in the development of these autoimmune diseases.

## Introduction

Autoimmune diseases can affect any organ and are classified into two categories: organ-specific diseases (e.g., autoimmune blistering disease (AIBD), type I diabetes, etc.) and systemic diseases (e.g., systemic lupus erythematosus (SLE), dermatomyositis (DM), etc.) and are associated with autoantibody production^1^. SLE usually manifests as constitutional symptoms and mucocutaneous initial symptoms and affects various organs, including the kidney, brain, heart, and lung, with specific autoantibodies, such as anti-double stranded DNA (dsDNA), nucleosome, Sm, histone, C1q, ribosomal P, Ro/SSa, La/SSb, and ribonucleoprotein (RNP) antibodies (Abs)^2,3^. Skin lesions occur in as many as 75–80% of SLE patients^4^. The lesions are categorized as acute (malar rash), subacute (papulosquamous lesions, lupus tumidus and chilblain lupus), chronic (discoid lupus), and bullous LE. Although acute cutaneous lupus is nearly always associated with systemic lupus, discoid lupus is infrequently (3–5%) associated with systemic disease^3^. DM is an idiopathic inflammatory myopathy with interstitial pneumonia and characteristic cutaneous manifestations, accompanied by specific autoantibodies such as anti-TIF1-γ, NXP2, MDA5, SAE, Mi2, and ARS Abs^2,5^. Pemphigus vulgaris (PV) and bullous pemphigoid (BP) represent two major AIBD disorders and affect only the skin and mucosa^6^. Autoantibodies, anti-desmoglein (Dsg)1 and Dsg3 Abs are seen in PV, whereas type XVII collagen at the dermo-epidermal junction and anti-BP180 and BP230 Abs are detected in BP^7^.

Type I interferon (IFN) is reportedly upregulated in autoimmune diseases, such as SLE and DM^8,9^. Type I IFN comprises 13 IFN-α subtypes, 2 IFN-β subtypes, and less-studied subtypes, such as IFN-ε, IFN-κ, and IFN-ω^10,11^. The in vitro antiviral and antiproliferative potencies vary among these subtypes, which may be due to their distinct affinities for the subunits of IFN-α/β receptor (IFNAR) 1 and 2^12,13^. Although plasmacytoid dendritic cells (pDCs) produce the majority of type I IFNs upon viral infection, all cell types can secrete type I IFNs^14^. Type II IFN, such as IFN-γ, is secreted from T lymphocytes and natural killer (NK) cells^15^. Type III IFNs, IFN-λ1, IFN-λ2, IFN-λ3, and IFN-λ4, are antiviral cytokines. Their functions are similar to those of type I IFNs. The binding of either a type I IFN to the IFNAR1/2 complex or a type III IFN to the IL-10R2/IFNLR1 complex triggers the activation of Janus kinase 1 (JAK1) and tyrosine kinase 2 (TYK2)^16^. This leads to the recruitment and phosphorylation of signal transducers and activators of transcription 1 and 2 (STAT1 and STAT2). STAT1 and 2 form a heterodimer that recruits IFN-regulatory factor 9 (IRF9) to form IFN-stimulated gene factor 3 (ISGF3)^17,18^. The binding of type II IFN dimers to the IFN-γ receptor 1 and 2 (IFNGR1/2) complex leads to the activation of JAK1 and JAK2, which subsequently phosphorylate STAT1. Phosphorylated STAT1 homodimers form the IFN-γ activation factor (GAF)^19^. Both ISGF3 and GAF induce the expression of genes regulated by IFN-stimulated response elements (ISREs) and gamma-activated sequence (GAS) promoter elements, respectively, resulting in the expression of IFN-stimulated genes (ISGs)^20^. Dysregulation of various IFNs may contribute to autoimmune pathogenesis^21^. In SLE, dysregulated IFN-γ is associated with autoantibody positivity, and upregulation of IFN-α/β activity can be observed in a pre-disease state^9,22^. Since dysregulation of various IFNs is seen in autoimmune diseases, the IFN and related gene expression profiles in peripheral blood can be useful in understanding the pathogenesis.

Retrotransposons are transposable elements involved in the pathogenesis of autoimmune diseases^23,24^. They are divided into two groups: those with long terminal repeats (LTRs) and non-LTRs^25^. LTR retrotransposons are recognized as endogenous retroviruses (ERVs) derived from exogenous retroviruses. In particular, the HERV-K virus is the youngest human endogenous retrovirus and is integrated almost solely as complete proviral copies in the human genome. Among non-LTR retrotransposons, the long interspersed nuclear element-1 (LINE-1) autonomous family of retroelements is active in mammalian genomes. It comprises approximately 17% of the human genome, yielding one-half million copies, and the transcription of LINE-1 is normally suppressed by the methylation of the respective promoter. Transcribed *LINE-1* induces the production of type I IFNs^26^. *Alu* sequences are short interspersed elements (SINEs) composed of two monomers linked by an A-rich linker sequence (A_5_TACA_6_). SINE-VNTR-Alu (SVA) is a composite element comprising an *Alu*-like CCCTCT hexamer region, a region of variable numbers of tandem repeats (VNTRs), and an HERV-K-derived region known as SINE-R. While all non-LTR retrotransposons share a poly-A tail, they are otherwise structurally different^25^.

We have investigated the roles of retrotransposons in the pathogenesis of autoimmune diseases, especially in SLE and DM. Furthermore, we examined whether type I IFN production enhances the JAK-STAT signaling pathway and induces ISGs using patient clinical samples and established human cell lines.

## Results

### Upregulation of retrotransposons with reduced LINE-1 promoter methylation in AIBD and DM

**Figure 1:**
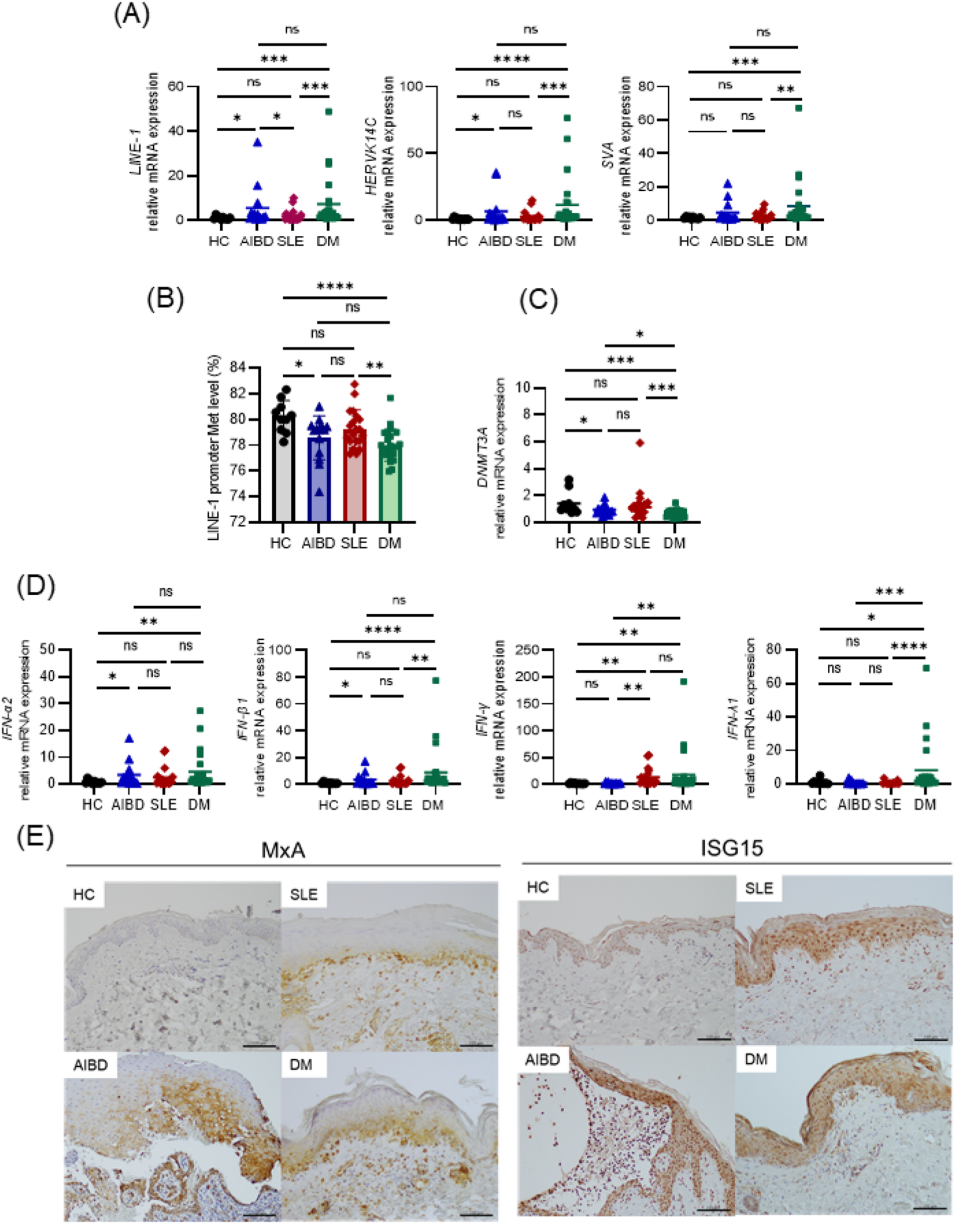
Different regulation of retrotransposons, IFNs, and ISGs among autoimmune diseases. (**A**) Retrotransposons are upregulated in AIBD and DM but not in SLE. The mRNA levels of retrotransposons, such as *LINE-1*, *HERVK14C*, and *SVA*, in the whole blood cells from HCs and patients with AIBD, SLE, or DM were analyzed by qPCR. (**B**) Reduced *LINE-1* promoter methylation in AIBD and DM. Methylation in the *LINE-1* promoter region was analyzed by pyrosequencing. (**C**) Reduced expression of *DNMT3A* in AIBD and DM. The mRNA levels of *DNMT3A* in blood cells were analyzed by qPCR. (**D**) Different induction of IFNs in autoimmune diseases. The mRNA levels of *IFN-α2*, *IFN-β1, IFN-γ,* and *IFN-λ1* were quantified by qPCR. (**A**–**D**) Data are shown as the means ± SD by Mann–Whitney test. HC (n = 10), AIBD (n = 14), SLE (n = 19), and DM (n = 24). *P < 0.05, **P < 0.01, ***P < 0.001, ****P < 0.0001, ns; not significant. (**E**) Increased expression of type I IFN-inducible proteins in skin lesions from autoimmune patients. Specimens from HC and skin lesions obtained from pretreated AIBD, SLE, and DM patients were stained with anti-MxA and anti-ISG15 antibodies. *Bars* = 100 μm.

To assess the involvement of retrotransposons in autoimmune diseases, we initially performed qPCR analyses of *LINE-1*^26^, *HERVK14C*, and *SVA* using peripheral blood cells from healthy controls (HCs) and patients with AIBD, SLE, or DM (Fig. 1A). Characteristically, the expression of all retrotransposon mRNAs was significantly upregulated in DM (*LINE-1*, HC vs. DM, *P* < 0.001; *HERVK14C*, HC vs. DM, *P* < 0.0001; and *SVA*, HC vs. DM, *P* < 0.001), and the expression of *LINE-1* was increased in AIBD (*LINE-1*: HC vs. AIBD, *P* < 0.05). In contrast, no statistically significant difference was detected between HC and SLE. Since methylation of the *LINE-1* promoter region plays a major role in *LINE-1* transcriptional regulation^27^, we analyzed the methylation levels of three promoter sites of *LINE-1* by pyrosequencing (Fig. 1B). The methylation level of the *LINE-1* promoter was significantly reduced in AIBD and DM patients but not in SLE patients, in comparison to that in HCs (HC vs. AIBD, *P* < 0.05; HC vs. DM, *P* < 0.0001). DNA methylation is generally catalyzed by DNA methyltransferase (DNMT) 1, DNMT3A, DNMT3B, lymphoid-specific helicase (LSH), and methyl-CpG-binding protein 2 (MeCP2), and mammalian DNA methylation patterns are mainly generated by DNMT3A and DNMT3B^28^. We determined that the mRNA expression level of *DNMT3A*, but not that of *DNMT1*, *DNMT3B*, *LSH* or *MeCP2*, was selectively downregulated in AIBD and DM compared to that in HC and SLE (HC vs. AIBD, *P* < 0.05; HC vs. DM, *P* < 0.001) (Fig. 1C and S1A). Collectively, these results suggested that hypomethylation of the *LINE-1* promoter by reduced expression of *DNMT3A* is likely to upregulate the expression of retrotransposons in DM and AIBD.

### Differential expression of IFNs in autoimmune diseases

To investigate the relationship between retrotransposons and interferonopathy, we next assessed the mRNA levels of *IFN-α2*, *IFN-β1* (type I)*, IFN-γ* (type II), and *IFN-λ1* (type III) in the patients’ peripheral blood cells (Fig. 1D and S1B). Notably, the mRNA levels of the type I-III IFNs examined were significantly upregulated in the DM patients (*IFN-α2*, HC vs. DM, *P* < 0.01; *IFN-α4*, HC vs. DM, *P* < 0.05; *IFN-β1*, HC vs. DM, *P* < 0.0001; *IFN-γ*, HC vs. DM, *P* < 0.01; and *IFN-λ1*, HC vs. DM, *P* < 0.05). In contrast, only type II IFN (*IFN-γ*) was upregulated in the SLE patients (*IFN-γ*, HC vs. SLE, *P* < 0.01), and only type I IFNs (*IFN-α2* and *IFN-β1*) were upregulated in AIBD (*IFN-α2*, HC vs. AIBD, *P* < 0.05, *IFN-β1*, HC vs. AIBD, *P* < 0.05)*.* Thus, these results clearly indicate that the induction spectra of *IFN* genes are distinct among DM, SLE, and AIBD patients.

To further assess the expression of ISGs in patients’ pretreated skin lesions (erythema, vesicle or erosion), we performed immunohistochemistry to detect type I IFN-inducible proteins such as MxA and ISG15. The MxA protein was clearly observed in the keratinocytes of the SLE (10/11), AIBD (7/9) and DM (3/3) samples but not in the skin samples of the HCs (0/3) (Fig. 1E and Table S1). The signal from the ISG15 protein was also strong in the cytoplasm of keratinocytes in the SLE (10/10), AIBD (9/9), and DM (3/3) samples. In contrast, skin samples of the HCs showed only nuclear staining of ISG15 (Fig. 1E and Table S1). These results indicated that the expression of IFN-inducible proteins is upregulated in the skin lesions of pretreated patients with DM, SLE, and AIBD. Although little is known about the roles of type I IFN in AIBD, the increased expression of IFN-inducible proteins in the pretreated skin lesions of AIBD patients suggests that IFNs may play suppressive or promoting roles in blister formation.

### Differential induction of IFN signaling factors in autoimmune diseases

**Figure 2:**
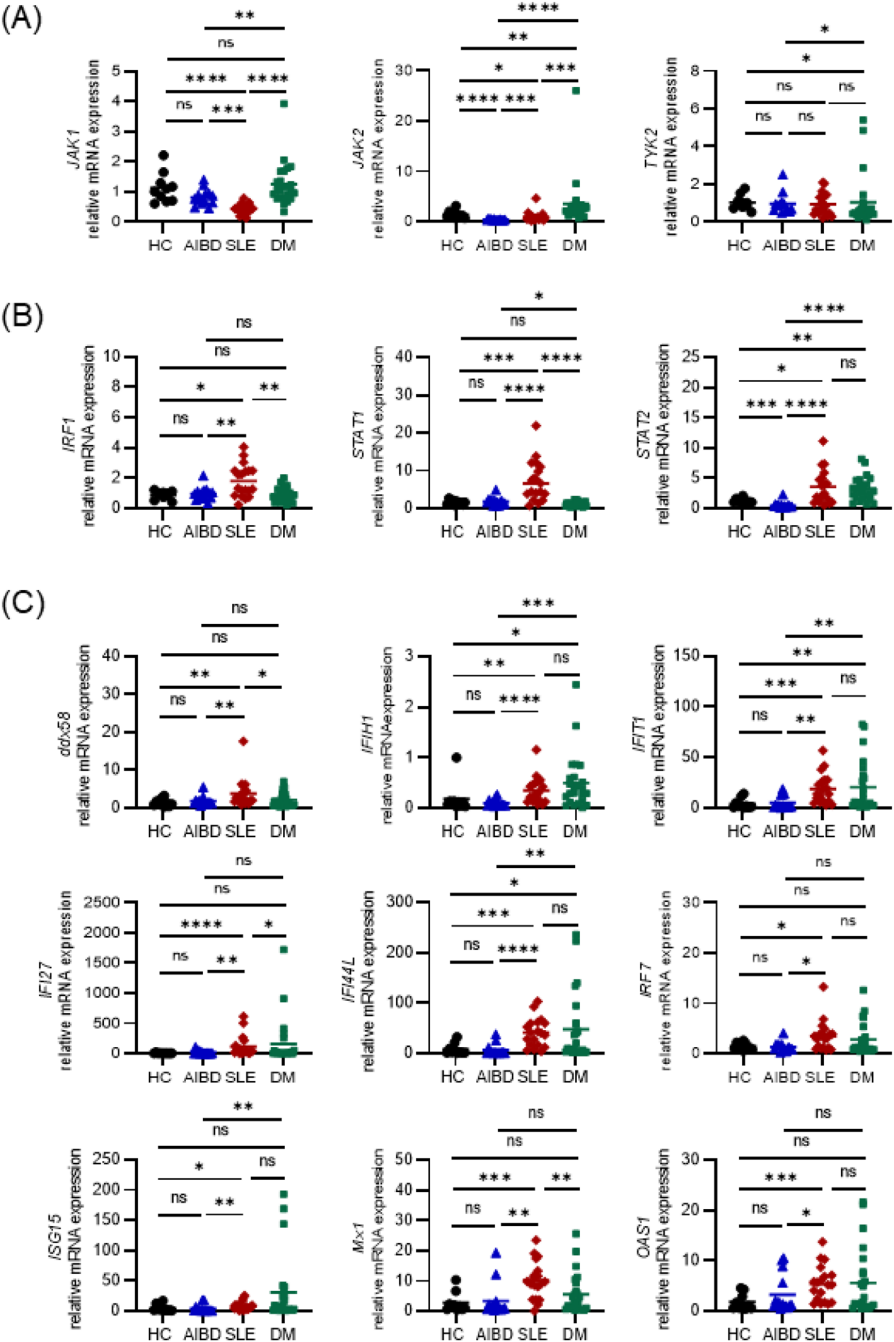
Diversity in the expression of IFN signaling factors in autoimmune diseases. (**A**) The expression level of nRTKs is upregulated in DM. The mRNA levels of nRTKs, such as *JAK1*, *JAK2*, and *TYK2,* in the whole blood cells from HCs and patients with autoimmune diseases were analyzed by qPCR*.* (**B**) Transcription factors are highly expressed in SLE. The mRNA levels of *STAT1*, *STAT2*, and *IRF1* were analyzed by qPCR. (**C**) ISGs are upregulated in SLE and DM but not in AIBD and HC. The mRNA levels of ISGs were analyzed by qPCR. (**A**–**C**) Data are shown as the means ± SD by Mann–Whitney test. HC (n = 10), AIBD (n = 14), SLE (n = 19), and DM (n = 24). *P < 0.05, **P < 0.01, ***P < 0.001, ****P < 0.0001, ns; not significant.

To clarify the gene expression in the IFN receptor signaling pathway, we performed qPCR analyses of *JAK1* and *JAK2*, *TYK2*, *STAT1* and *STAT2*, and *IRF1*. Compared with that of HCs, the mRNA level of *JAK2* was decreased in AIBD patients (HC vs. AIBD, *P* < 0.0001), and the levels of *JAK1* and *JAK2* were also decreased in SLE patients (*JAK1*, HC vs. SLE, *P* < 0.0001; *JAK2*, HC vs. SLE, *P* < 0.05). In contrast, *JAK2* upregulation and *TYK2* downregulation were detected in DM samples (*JAK2*, HC vs. DM, *P* < 0.01; *TYK2*, HC vs. DM, *P* < 0.05) (Fig. 2A). Moreover, the expression levels of *IRF1, STAT1, and STAT2* were significantly increased in the SLE samples (*IRF1*, HC vs. SLE, *P* < 0.05; *STAT1*, HC vs. SLE, *P* < 0.001; *STAT2*, HC vs. SLE, *P* < 0.05), although an increase in *STAT2* expression alone was detected in the DM samples (HC vs. DM, *P* < 0.01) (Fig. 2B). We further examined the expression of downstream IFN-stimulated genes (ISGs), such as *ddx58, IFIH1, IFIT1, IFI27, IFI44L, IRF7, ISG15, Mx1,* and *OAS1,* as well as IFN-γ-specific downstream chemokines *CXCL9/MIG* and *CXCL10/IP-10*, by qPCR (Fig. 2C and S1C). Notably, the expression levels of all ISGs were significantly increased in the SLE patients compared to those of the HCs, while increased expression levels of *IFIH1, IFIT, IFI44L, and CXCL-10* were detected in the DM patients compared to the HCs. In contrast, no significant induction of ISGs was detected in the AIBD patients.

Collectively, the data showed that type I-III IFNs are activated in DM. In contrast, in SLE, only type II IFN is activated, and transcription factors (TFs) and ISGs in type II IFN signaling pathway are also upregulated.

### Expression profiles of retrotransposons and IFNs in patients

Since distinct IFN expression was detected among the DM, SLE, and AIBD patients, we analyzed the expression patterns of retrotransposons and IFN pathways. We categorized retrotransposons and IFN-related genes into four categories: retrotransposons and IFNs, nonreceptor tyrosine kinases (nRTKs), TFs, and ISGs (Fig. S2). Patients SLE03, SLE04, DM03, DM05, DM05, DM23, DM24, and AIBD04 showed overexpression of retrotransposons and type I and/or II IFNs but no consistent upregulated expression of nRTKs, TFs or ISGs. In addition, high induction of TFs, particularly *STAT1* and *STAT2,* was detected in the SLE patients, whereas *STAT2* was selectively induced in the DM patients (Fig. S2). Although high ISG induction was commonly found in —SLE patients, several patients, including AIBD06, AIBD13, DM02, DM17, DM18, DM21, and DM22, also showed high expression of some ISGs (Fig. S2). Taken together, these data showed that there was no significant correlation between IFNs and ISGs.

### Correlation of IFNs and retrotransposons with downstream signaling

**Figure 3:**
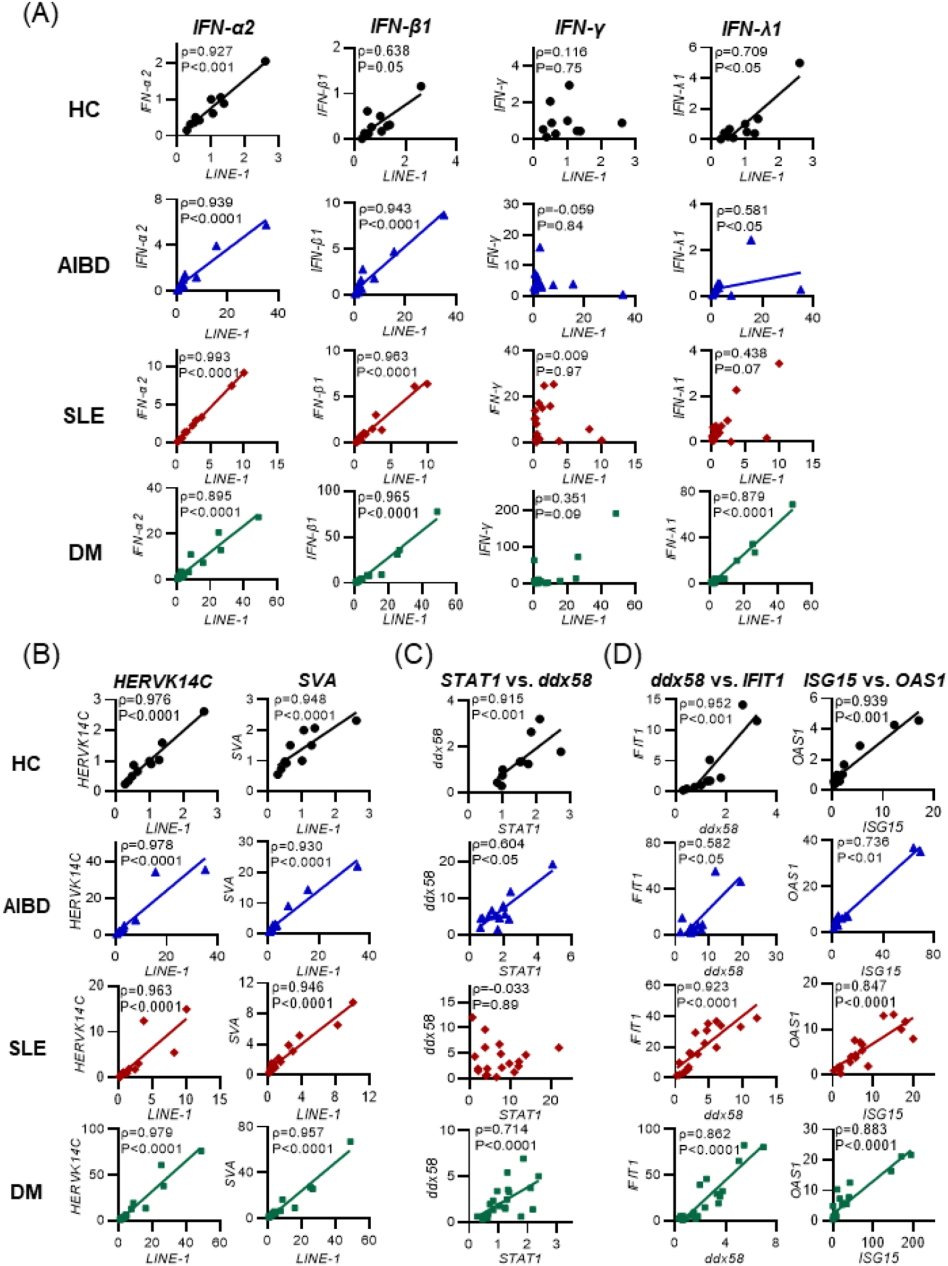
Correlation of mRNA levels of retrotransposons, IFNs, STAT1, and ISGs in autoimmune diseases. (**A**) Type I and III IFNs, but not type II IFN, are correlated with *LINE-1*. The correlation of mRNA levels between *LINE-1* and IFNs, such as *IFN-α2*, *IFN-β1, IFN-γ,* and *IFN-λ1*, was analyzed. (**B**) Synchronized expression of *LINE-1* and retrotransposons. The correlations of the mRNA levels between *LINE-1* vs. *HERVK14C* and *SVA* retrotransposons are shown. (**C**) There was no positive correlation between *STAT1* and *ddx58* in SLE. The correlation of mRNA levels between *STAT1* and *ddx58,* an ISG, was examined. (**D**) Positive correlation in ISGs. The correlations of mRNA levels between ISGs, such as *ddx58* with *IFIT1* and *ISG15* with *OAS1,* were analyzed. (**A**–**D**) Representative correlation coefficients are shown between two genes in each group, and ρ-values were determined by nonparametric Spearman’s rank correlation test. The numbers of samples: HC (n = 10), SLE (n = 19), AIBD (n = 14), and DM (n = 24).

To evaluate the relationship between retrotransposons and IFNs, we performed Spearman’s rank correlation analysis. The correlations of *LINE-1* with IFNs, nRTKs, TFs, and ISGs were analyzed in patients with DM, SLE or AIBD and HCs. First, we tested the correlation of retrotransposons with type I-III IFNs (Fig. 3A). Significant positive correlations between *LINE-1* and type I IFNs were detected in DM samples (*IFN-α2,* ρ = 0.895, *P* < 0.0001; *IFN-β1,* ρ = 0.965, *P* < 0.0001), SLE samples (*IFN-α2,* ρ = 0.993 *P* < 0.0001; *IFN-β1,* ρ = 0.963, *P* < 0.0001), and AIBD samples (*IFN-α2,* ρ = 0.939, *P* < 0.0001; *IFN-β1,* ρ = 0.943, *P* < 0.0001). *LINE-1* also showed positive correlations with *IFN-λ1* in the HCs, AIBD patients and DM patients but not in the SLE patients. In contrast, *LINE-1* showed no correlation with type II *IFN-γ* in patients with autoimmune diseases or HCs. Furthermore, we detected coordinated relationships among retrotransposons, such as *LINE-1* and *HERVK14C* or *SVA*, in each type of autoimmune disease and HC sample (Fig. 3B). Similar to the correlation of *LINE-1* and *IFN-λ1,* the correlation between *STAT1* and *ddx58* showed significant positive correlations in the DM, AIBD, and HC samples but not in the SLE samples (Fig. 3C). Among ISGs, both *ddx58* with *IFIT1* and *ISG15* with *OAS1* showed significant positive correlations in all types of autoimmune disease and HC samples (Fig. 3D).

**Figure 4:**
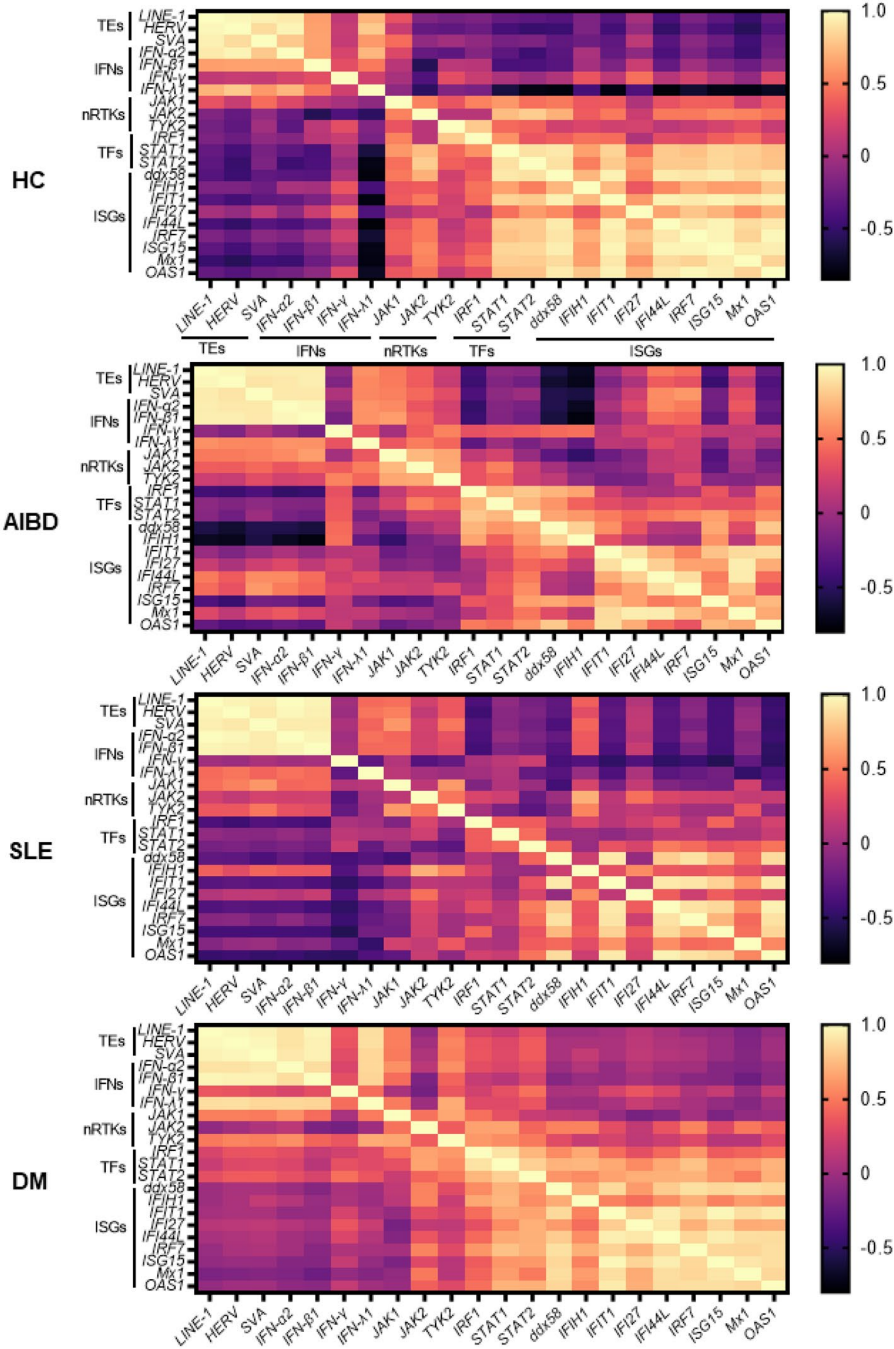
Correlation heat map of transcripts in HCs and patients with autoimmune diseases. A hierarchically clustered heat map matrix showing the correlation between the expression of each gene. The ρ-values, which were calculated by the nonparametric Spearman’s rank correlation test, are represented as colored bars.

The correlation of each gene in the autoimmune disease and HC samples is summarized in a correlation heat map (Fig. 4 and Tables S2–S5). Retrotransposons and type I IFN showed a clear, positive correlation regardless of disease. On the other hand, the correlation of retrotransposons and nRTKs was different depending on the disease. Retrotransposons showed significant positive correlations with *JAK1* and *TYK2* in DM samples and only *JAK1* in SLE samples, not in AIBD or HC samples (Tables S2–S5). Although retrotransposons did not correlate with TFs or ISGs, they showed coordinated expression with each gene, especially those expressed in HC and DM (Fig. 4). These results strongly suggested that retrotransposons and type I IFN production are positively synchronized; however, retrotransposon expression is not positively correlated with IFN receptor downstream signaling, except for that of some nRTKs.

### Mutual regulation of retrotransposons and IFNs in cultured cells

**Figure 5:**
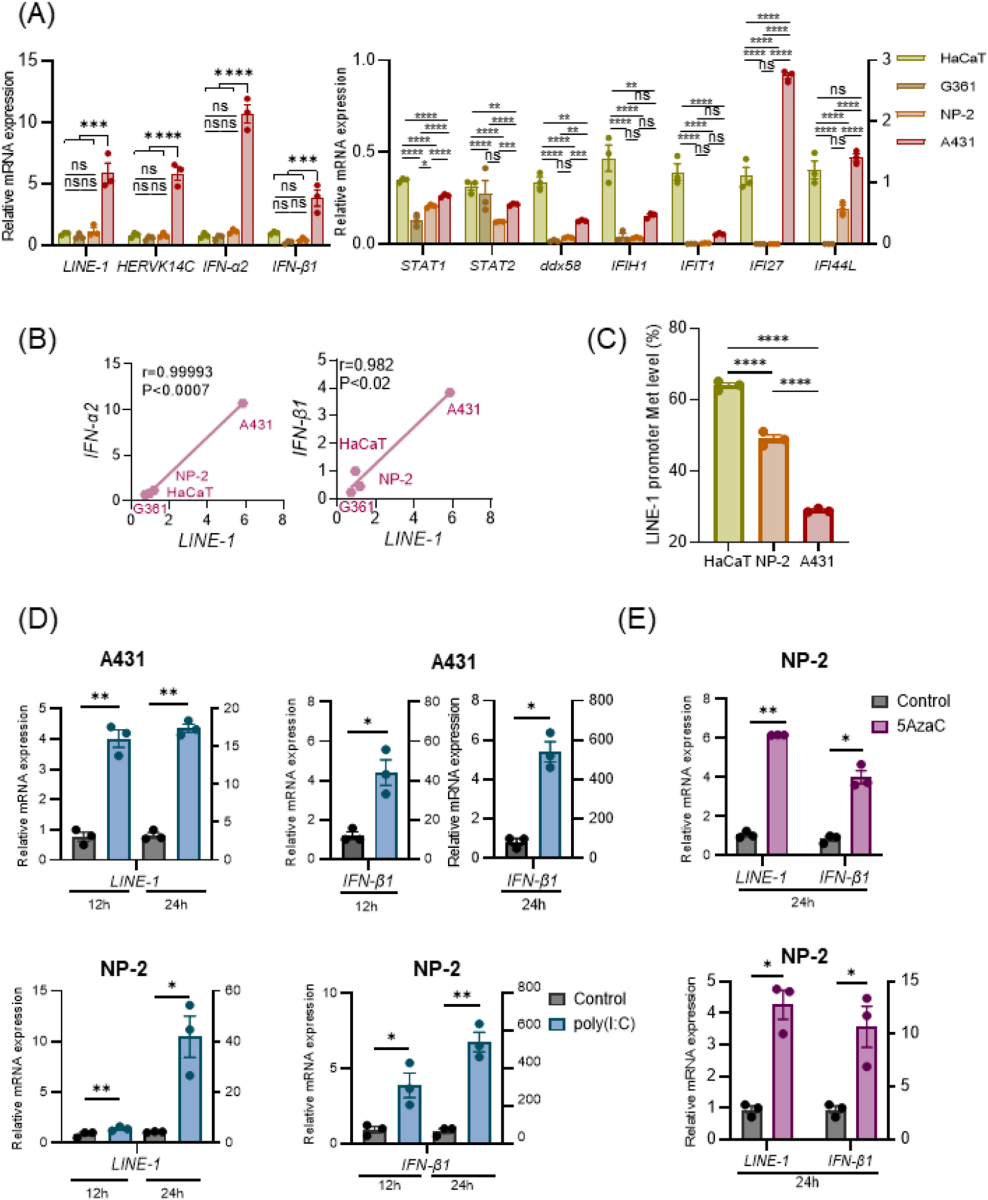
Correlated expression of *LINE-1* and type I IFN in cell lines. (**A**) Retrotransposons and type I IFNs are upregulated in A431 cells. The mRNA levels of retrotransposons*,* TFs, and ISGs in the indicated cell lines were examined by qPCR. (**B**) Positive correlation of *LINE-1* and IFNs in cell lines. The correlation of the relative mRNA levels between *LINE-1* vs. *IFN-α2* or *IFN-β1* was analyzed, and Pearson correlation coefficients (*r*-values) are shown. (**C**) Methylation in the *LINE-1* promoter was reduced in A431 cells. The methylation levels in three sites of the *LINE-1* promoter were analyzed by pyrosequencing. (**D**) Poly(I:C) induces the expression of *LINE-1* and *IFN-β1.* A431 and NP-2 cells were treated with 30 ng/ml poly(I:C) for 12 and 24 h, and the relative mRNA expression levels of *LINE-1* and *IFN-β1* were examined by qPCR. (**E**) Hypomethylation enhances *LINE-1* and *IFN-β1.* A431 and NP-2 cells were treated with 100 μM 5AzaC for 24 h, and the expression levels of *LINE-1* and *IFN-β1* were examined by qPCR. Data are shown as the mean ± SEM by one-way ANOVA with Tukey’s post hoc test (**A**,**C**) or Student’s *t*-test (**D**,**E**), n = 3. *P < 0.05, **P < 0.01, ***P < 0.001, ****P < 0.0001, ns; not significant.

Finally, we investigated the correlation between IFNs and retrotransposons in vitro using four established human adherent cell lines, NP-2, A431, G361, and HaCaT cells. Interestingly, enhanced expression levels of *LINE-1*, *HERVK14C*, *IFN-α2,* and *IFN-β1* were detected in A431 cells (Fig. 5A). Furthermore, relatively high expression levels of TFs and ISGs were detected in HaCaT and A431 cells but not in NP-2 or G361 cells. The expression of *LINE-1* and *IFN-α2* or *IFN-β1* was positively correlated in HaCaT and A431 cells (Fig. 5B). The methylation level of the *LINE-1* promoter, which was analyzed by pyrosequencing, was significantly reduced in A431 cells compared to that in NP-2 and HaCaT cells (Fig. 5C).

Poly(I:C), a synthetic analog of double-stranded RNA, is a potent inducer of type I IFNs^29,30^. Upon stimulation with poly(I:C), the expression of *LINE-1*, as well as *IFN-β1*, was remarkably increased in A431 and NP-2 cells (Fig. 5D). 5AzaC, a demethylation agent, causes hypomethylation and induction of *LINE-1*^31^. Treatment with 5AzaC elevated the mRNA levels of both *LINE-1* and *IFN-β1* in A431 and NP-2 cells (Fig. 5E). These results revealed the mutual regulation of *LINE-1* and type I IFN in cultured cells. Finally, the coordinated expression of retrotransposons and type I IFN was observed not only in the blood cells of patients receiving treatment but also in adherent culture cells in vitro.

## Discussion

In this study, we detected elevated levels of type I IFN mRNA in blood samples of patients with DM and AIBD. Interestingly, the expression of retrotransposons and type I IFNs showed synchronicity regardless of disease, suggesting that various pathways of type I IFN activation may be mutually correlated with retrotransposon activation. In DM and AIBD, the type I IFN pathway may be continuously activated at a low level in patients undergoing treatment (Figs. 1D and 2). Our results suggested that the altered expression of retrotransposons may induce constitutive type I IFN signaling prematurely, and additional factors, such as viral infections, may further upregulate the type I IFN pathway^32^, leading to the development of autoimmune diseases. In contrast, type II IFN activation was not correlated with retrotransposon expression (Figs. 3 and 4), indicating that *IFN-γ* expression is regulated by a different mechanism than that involved in upregulated retrotransposon expression. In addition to IFNs, the expression of various ISGs was coordinated; however, their expression was not correlated with that of IFNs (Fig. 4). A time lag between IFN production and receptor binding or prolonged expression of ISGs due to unphosphorylated STAT1^33^ or positive feedback stimulated by *STAT1* expression^34^ may account for this discrepancy. In addition, IFN-independent constitutive upregulation of STAT1/2 and IRF1 expression has been reported^35^. Phosphorylation was not required to realize this increase, but only protein expression was observed. A protective role against viral infection has also been reported for STAT1/2 and IRF1^36^; these findings have not yet been reported in SLE or DM.

We showed the mutual regulation of *LINE-1* and IFN using cultured cells, and this finding was compatible with the data obtained with the clinical samples. The number of *LINE-1* mRNA transcripts was increased in the kidneys of patients with lupus nephritis and in the minor salivary glands of patients with primary Sjögren’s syndrome, in concert with type I IFN expression^23^. Moreover, *LINE-1* RNA contributes to the production of type I IFN through both the MDA5 and RIG-I pathways^26^, and *LINE-1* induces type I IFN expression, which in turn regulates the activity and propagation of *LINE-1*^37^. Thus, *LINE-1* and type I IFN seem to be mutually regulated.

**Figure 6:**
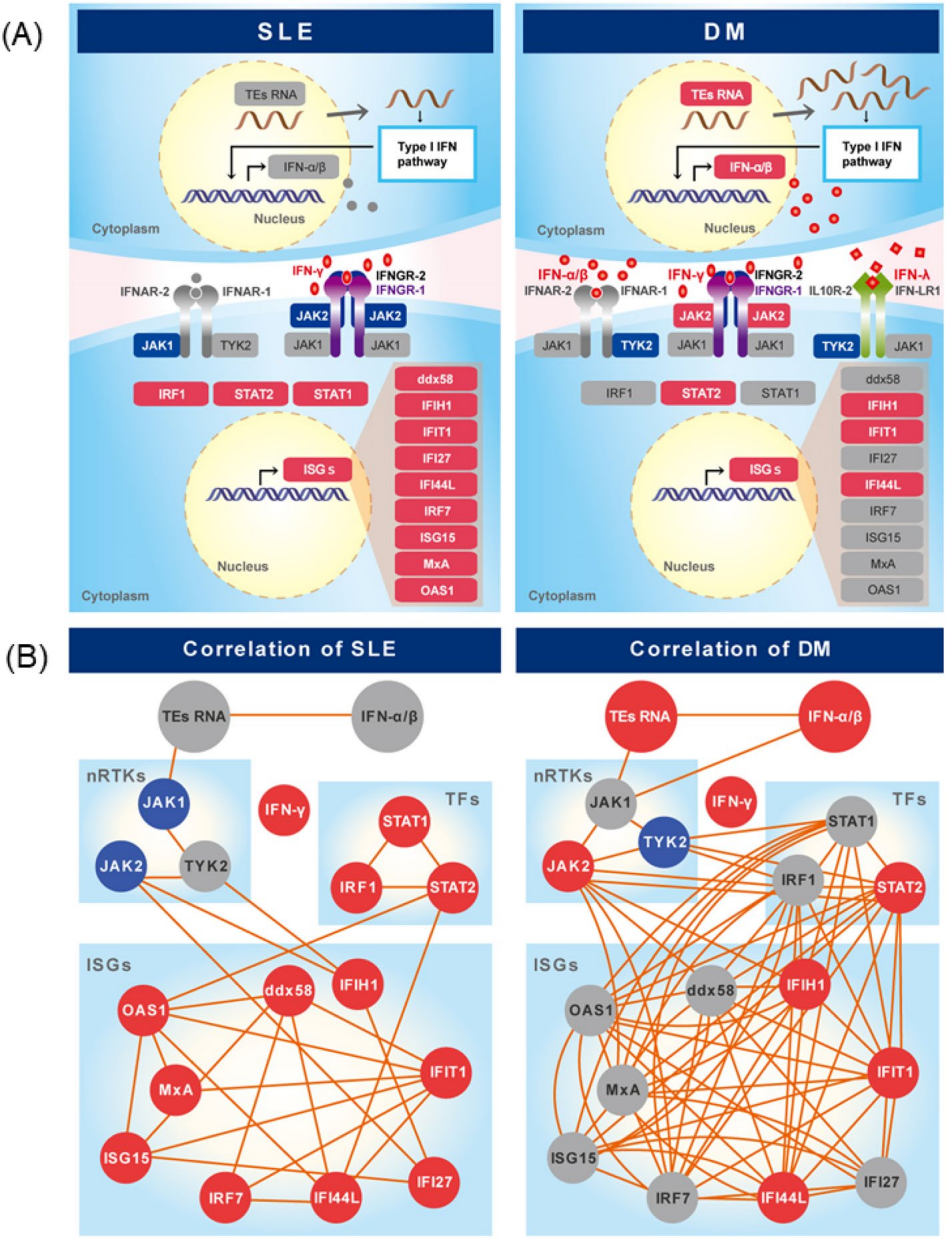
Scheme of retrotransposon-mediated IFN responses in SLE and DM. (**A**) The activation level of the IFN production pathway in patients with SLE and DM differed. Genes with significantly elevated and suppressed expression compared with those in the control group are highlighted in *red* and *blue*, respectively. (**B**) The correlation among genes. Genes with significantly elevated and suppressed expression compared with those in the control are highlighted in *red* and *blue*, respectively. The connected lines show the significant positive correlations between two genes calculated by a nonparametric Spearman’s rank correlation test. TE; transposable element, nRTK;: nonreceptor tyrosine kinase, TF; transcription factor.

We showed the distinct expression patterns of retrotransposons and IFN-related genes between SLE and DM patients. The significant positive correlation between retrotransposons and type I IFN and the absence of a positive correlation between retrotransposons and IFN receptor downstream signaling, except for some nRTKs, are common to both SLE and DM. The difference between SLE and DM relates to INF expression; only type II IFN and *IFN-γ* expression was upregulated in SLE, and in contrast, the expression of all types of IFNs was upregulated in DM (Figs. 1D and 6A). In downstream signaling induced by the IFN receptor, retrotransposons showed significant positive correlations with *JAK1* and *TYK2* expression in DM and only with *JAK1* expression in SLE. The expression of TFs and ISGs was upregulated in SLE, but only *STAT2* and some ISGs *(IFIH1*, *IFIT1*, and *IFI44 L)* expression was upregulated in DM. In contrast, comparing the correlation of each gene, larger numbers of positive correlations were seen among genes in DM (Fig. 6B, Tables S4 and S5). ISGs were generally correlated with TFs in DM. Although *STAT1* and *IRF1* expression was were upregulated in SLE, no significant correlations were seen between *STAT1* and ISGs or between *IRF1* and ISGs (Fig. 6B). High *STAT1* expression is a characteristic of SLE^38^. In accordance with our findings, Kartonitsch et al*.* suggested that the increased level of the STAT1 protein was involved in *IFN-γ* responsiveness and resulted in chronically activated IFN-γ signaling in SLE^39^. Subsequently, IFN-γ and downstream chemokines, including CXCL9 and CXCL10, are dysregulated prior to disease flares in SLE patients^40–43^. However, our results show no dysregulation in the expression of CXCL9 or CXCL10 from mRNA level in whole blood cells.

The expression of *JAK1* and *JAK2* in SLE and *TYK2* in DM was significantly downregulated, which might be due to the negative feedback of upregulated TFs. On the other hand, upregulation of *LINE-1* and type I IFNs but no significant upregulation of the downstream components of the IFN pathway were observed in AIBD, which may reflect the fact that AIBD is an organ-specific autoimmune disease.

There are several clinical implications of this study. We noticed that the SLE flare was more frequent in the high LINE-1 and IFN groups (SLE Pt 3, 4, 7, 18 in Fig. S2 and Table S7), while it was not clear in the DM group. This result is consistent with the high prevalence of p40, encoded by LINE-1, in serum correlating with flares and high type I IFN scores in SLE^44^. On the other hand, SLE and DM samples showed similar but distinct cutaneous manifestations. The triggers for skin lesions depend on the disease: in DM, external stimuli (Köbner’s phenomenon) and, in SLE, sun exposure cause inflammation of the skin and infiltration of peripheral blood leukocytes that may secrete IFNs. The epidermis is thought to respond to these IFNs and produce ISGs. As shown in Fig. 1E, MxA expression was especially prominent in the basal layer of the epidermis, suggesting that peripheral blood cells infiltrating the epidermal-dermal interface influence MxA expression in the epidermis. Although we found no clear association between the expression of LINE-1/IFNs and cutaneous manifestations, we speculate that circulating peripheral blood cells may be involved in the skin rash: peripheral blood cells in SLE and DM secrete different cytokines and may cause different skin lesions.

Recently, antibody drugs such as anifrolumab against IFNAR and BIIB059 against the BDCA-2 antigen in pDCs have been developed to treat SLE^45^. Anifrolumab reduced the glucocorticoid dose and decreased the severity of skin disease in SLE patients^46^. Since *STAT1/2* are significantly upregulated in SLE samples, an inhibitor of the JAK-STAT pathway is another promising therapeutic candidate. In addition, the expression of multiple IFNs, *STAT2* and retrotransposons was upregulated in DM (Figs. 1A,D, 2B, and 6A), and JAK2 expression was high in DM and strongly associated with downstream TFs and ISGs (Fig. 6), suggesting that JAK2 inhibitors may be effective in DM. In this context, tofacitinib, a pan-JAK inhibitor, seems to be a possible therapeutic option for use against DM^47,48^. Although the mechanism by which retrotransposons induce the production of type I IFNs remains to be elucidated, inhibitors targeting retrotransposons and IFN interactions may be preferable drugs to treat DM.

In conclusion, our analyses of peripheral blood cells from SLE and DM patients receiving treatment revealed distinct expression patterns of retrotransposons and IFNs. The expression levels of retrotransposons and type I and III IFNs were generally correlated, and the upregulation of these genes may be partly responsible for the development of DM. Further investigations will clarify these relationships.

## Materials and methods

### Patients

Nineteen patients with SLE and 24 patients with DM were recruited from Gunma University. For comparison, we also investigated 14 patients with AIBD (bullous pemphigoid, 10; pemphigus vulgaris, 3; pemphigus foliaceus, 1) and 10 unaffected healthy controls. There was no racial difference among participants. Detailed information on the patients is shown in Supplementary Tables 6–8. We extracted RNA from peripheral blood cells from patients receiving treatment. The DM and healthy control samples (HC) were the same as those described previously^32^. We also collected biopsied skin samples from the lesions (erythema, vesicle and erosion) of 11 SLE patients, 9 AIBD patients, 3 DM patients, and 3 healthy individuals. Most of the patients under treatment had well controlled and stable disease. Informed consent was obtained from all subjects and/or their legal guardian(s). Ethics approval for this study was granted by the ethics commission of Gunma University (reference number: 1349) in accordance with the Declaration of Helsinki protocols.

### DNA and RNA extraction from peripheral blood cells

DNA was extracted from the patients’ whole blood cells using a Wizard Genomic DNA Purification kit (Promega) according to the manufacturer’s instructions. RNA was extracted from the patients’ peripheral blood cells as follows: whole blood collection was performed with EDTA-treated tubes. After washing the blood cells three times with phosphate-buffered saline (PBS), the red blood cells were lysed with ammonium–chloride–potassium (ACK) lysing buffer. RNA was extracted from white blood cells using an RNeasy Mini Kit (Qiagen). Complementary DNA (cDNA) was synthesized using GoScript Reverse Transcriptase (Promega).

### Detection of retrotransposons

LINE-1, human endogenous retrovirus (HERVK14C), and SVA were assessed using cDNA prepared from the patients’ blood samples by quantitative polymerase chain reaction (qPCR), as previously described^26,49^. To examine the expression of methylation enzymes, including IFNs, JAK family members, TFs, and ISGs, qPCR was performed using specific primer pairs (Table S9). In brief, qPCR was performed using a THUNDERBIRD Probe and SYBR qPCR Mix (Toyobo). The expression level relative to that of a housekeeping gene was used in the analyses. Real-time PCR was performed using an ABI 7300 PCR thermal cycler (Applied Biosystems) under the following conditions: 10 min at 95 °C, 40 cycles of 15 s at 95 °C, and 1 min at 60 °C. The data were analyzed using the ΔΔCt method.

### Immunohistochemistry

Immunohistochemistry (IHC) was performed as described^50,51^. Paraffin embedded biopsy skin specimens from 11 SLE patients, 9 AIBD patients, and 3 DM patients before treatment and 3 healthy individuals were available. These samples were obtained from the same individuals whose peripheral whole blood samples were studied. Deparaffinized and rehydrated sections were autoclaved in 5 mM citrate buffer for 10 min at 121 °C for antigen retrieval. Endogenous peroxidase activity was blocked with peroxidase blocking solution (DAKO) for 5 min. The sections were incubated overnight at 4 °C with an anti-MxA antibody (1/100, sc-166412, Santa Cruz Biotechnology) and an anti-ISG15 antibody (1/50, ab131119, Abcam), followed by incubation for 1 h with a secondary antibody solution (anti-mouse K4000 and anti-rabbit K4002 antibodies, respectively, DAKO). Nuclear staining was performed using hematoxylin. The specificity of staining was confirmed using a matched isotype normal IgG as the first antibody. Immunoreactivity was evaluated in comparison with the staining patterns of skin specimens from normal individuals. The proportion (%) of positive epidermal cells was evaluated in each sample, and the mean proportion was assessed as follows: > 50%, 3; 50–20%, 2; < 20%,1; -,0. The intensity was expressed semiquantitatively using a four-level ordinal scale: 0, negative; 1, low; 2, intermediate; and 3, high. The scoring was performed independently by two observers (Y.K. and A.S.) (Table S1).

### Quantification of L1 promoter methylation by pyrosequencing

A pyrosequencing-based methylation assay was used to assess the three CpG sites in the promoter region of *LINE-1*. To analyze *LINE-1* promoter methylation, genomic DNA was extracted from peripheral whole blood cells and modified with sodium bisulfite using an EpiTect bisulfite kit (Qiagen). PCR and subsequent pyrosequencing for *LINE-1* were performed with a PyroMark kit (Qiagen). The PCR products were immobilized and converted to the single-stranded form to act as templates in the pyrosequencing reaction using the Pyrosequencing Vacuum Workstation (Qiagen). The amount of C relative to the sum of the amounts of C and T at each CpG site was calculated. The average percentages of the relative amounts of C in the three CpG sites of *LINE-1* were used as the overall *LINE-1* methylation levels. The position was from 331 to 318 in GenBank accession number X58075. Samples were analyzed with a PyroMark Q24 system (Qiagen).

### Cell culture, real-time PCR, and reagents

A431 (epidermoid carcinoma), G361 (malignant melanoma), NP-2 (glioma), and HaCaT (epidermal keratinocytes) cells were cultured in DMEM containing 10% fetal bovine serum, 100 IU/ml penicillin G and 100 μg/ml streptomycin at 37 °C in a 5% CO_2_ atmosphere. Cellular RNA was extracted from each cell line using an RNeasy mini kit (Qiagen). Complementary DNA was prepared with GoScript Reverse Transcriptase (Promega) using random primers. qPCR was performed by using THUNDERBIRD Probe and SYBR qPCR Mix (Toyobo), as described above. The cells were treated with 100 μM 5-azacytidine (5AzaC) (Cayman Chemical) or 30 ng/ml poly(I:C) (InvivoGen) for 12 and 24 h, and RNA was then extracted.

### Statistical analyses

Two-group comparisons of continuous data were assessed using the Mann–Whitney U test because the data were not normally distributed. The correlations between the gene expression levels were determined using a nonparametric Spearman’s rank correlation test. The data are shown as the means ± SEM from experiments performed in triplicate, and *p* values were calculated using Student’s *t*-test or one-way ANOVA followed by post hoc Tukey HSD test for in vitro experiments. The correlations between the gene expression levels were determined using Pearson's correlation coefficient of in vitro experiments. *P* values < 0.05 were considered statistically significant.

## Supplementary Information

Supplementary Information.
