# Supplementary material for: Coordination of retrotransposons and type I interferon with distinct interferon pathways in dermatomyositis, systemic lupus erythematosus and autoimmune blistering disease
Source: Sci Rep. 2021 Nov 30;11:23146. doi: 10.1038/s41598-021-02522-6 (PMC8632942; doi:10.1038/s41598-021-02522-6)
Supplement: Supplementary file 1 — Supplementary Information. [file 41598_2021_2522_MOESM1_ESM.docx]

Supplementary File

**Coordination of retrotransposons and type I interferon with distinct interferon pathways in dermatomyositis, systemic lupus erythematosus and autoimmune blistering disease**

Yuko Kuriyama, M.D., Ph.D. ^1^, Akira Shimizu, M.D., Ph.D.^1,2*^, Saki Kanai^1^, Daisuke Oikawa, Ph.D.^3^, Sei-ichiro Motegi, M.D., Ph.D.^1^, Fuminori Tokunaga, Ph.D.^3^, and Osamu Ishikawa, M.D., Ph.D.^1^

^1^Department of Dermatology, Gunma University Graduate School of Medicine, Gunma, Japan.

^2^ Department of Dermatology, Kanazawa Medical University, Ishikawa, Japan.

^3^Department of Pathobiochemistry, Graduate School of Medicine, Osaka City University, Osaka, Japan.

**Contents:**

**-Supplementary figures and figure legends**

**-Supplementary tables**

A

B

C

**Supplementary Figure 1.** Expression levels of retrotransposons, DNA methylation-related genes, and type I IFN from the whole blood in autoimmune diseases. (**A**) *DNMT1* and *DNMT3B* are partially upregulated in autoimmune diseases. *DNMT1, LSH, MeCP2,* and *DNMT3B* in the blood cells were analyzed by qPCR. (**B**) *IFN-α4* is upregulated in DM. The mRNA level of *IFN-α4* in blood cells was analyzed by qPCR. (**C**) CXCL10 is upregulated in DM. The mRNA levels of *CXCL9* and *CXCL10* in blood cells were analyzed by qPCR. (**A**, **B, C**) Data are shown as the means ±SD by Mann–Whitney test. HC (n=10), AIBD (n=14), SLE (n=19), and DM (n=24). *P < 0.05, **P < 0.01, ***P < 0.001, ****P < 0.0001, ns; not significant. **
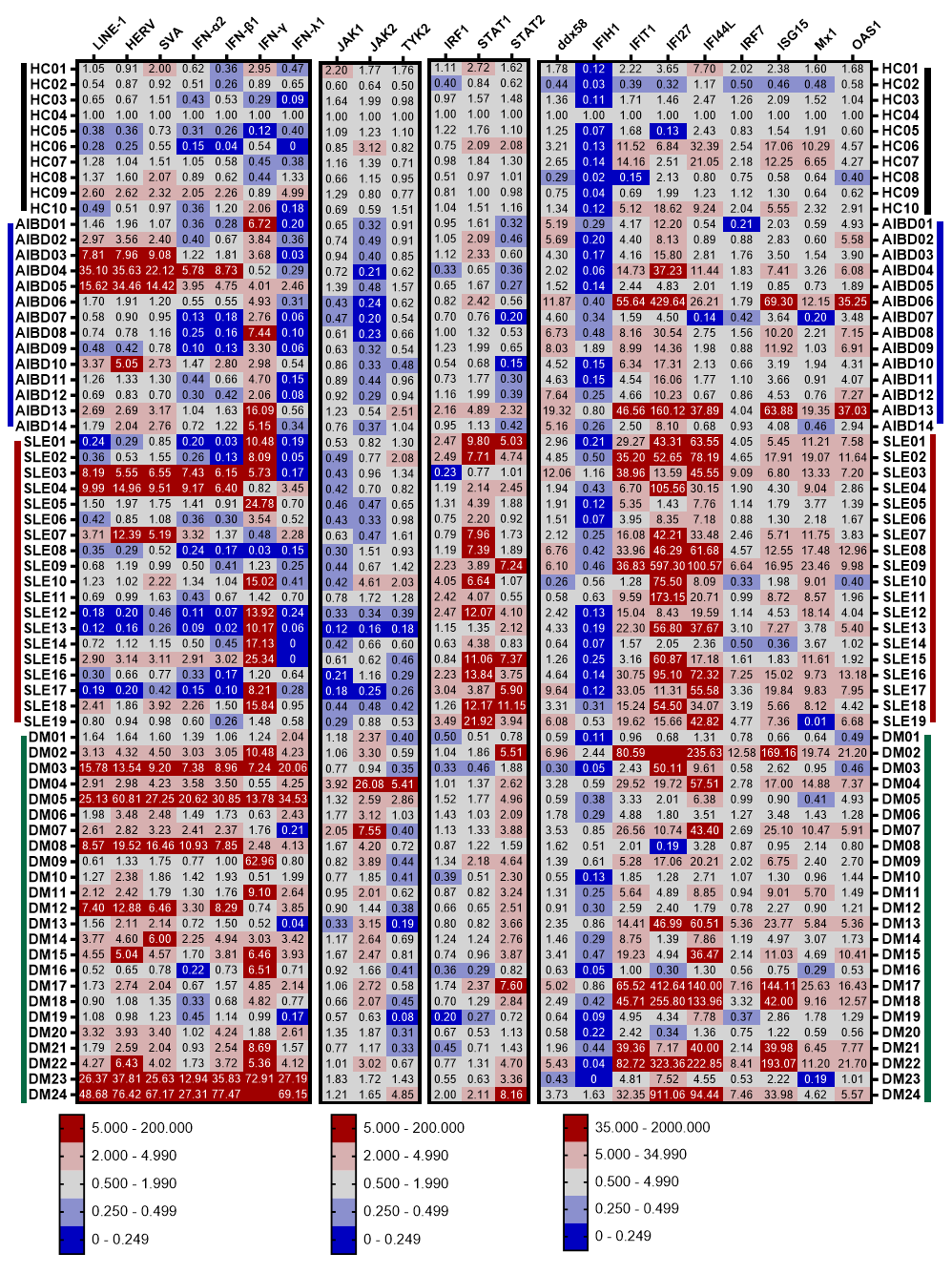
Supplementary Figure 2.** Gene expression profiles of all samples. Heat map matrix showing the mRNA levels of all samples. The mRNA levels of *LINE-1*, *HERVK14C*, *SVA*, *IFN-α2*, *IFN-β1, IFN-γ, IFN-λ1, JAK1*, *JAK2*, *TYK2, IRF1, STAT1, STAT2, ddx58, IFIH1, IFIT1, IFI27, IFI44 L, IRF7, ISG15, Mx1,* and *OAS1* were quantified by qPCR. HC (n=10), AIBD (n=14), SLE (n=19), and DM (n=24). Expression levels are represented by colored bars.

**Supplementary Table 1.** Evaluation of staining by anti-MxA and anti-ISG15 antibodies in skin lesions of autoimmune patients before treatment.

| MxA |  |  |  |  |
| --- | --- | --- | --- | --- |
|  | proportion | | | |
|  | >50% | 50–20% | 20–0% | 0% |
|  | 3 | 2 | 1 | 0 |
| HC (n=3) | 0 | 0 | 0 | 3 |
| SLE (n=11) | 3 | 6 | 1 | 1 |
| AIBD (n=9) | 1 | 6 | 0 | 2 |
| DM (n=3) | 3 | 0 | 0 | 0 |
|  |  |  |  |  |
| ISG15 |  |  |  |  |
|  | proportion | | | |
|  | >50% | 50–20% | 20–0% | 0% |
|  | 3 | 2 | 1 | 0 |
| HC (n=2) | 0 | 0 | 0 | 2 |
| SLE (n=10) | 9 | 1 | 0 | 0 |
| AIBD (n=9) | 9 | 0 | 0 | 0 |
| DM (n=3) | 3 | 0 | 0 | 0 |

**Supplementary Table 2.** Correlation between each gene in the HCs. The correlation coefficients in HCs (n=10) are shown between two genes, and ρ-values were determined by nonparametric Spearman’s rank correlation test. P values <0.05 were considered statistically significant and are shown in bold.

**Supplementary Table 3.** Correlation between each gene in AIBD. The correlation coefficients in AIBD (n=14) are shown between two genes, andρ-values were determined by nonparametric Spearman’s rank correlation test. P values <0.05 were considered statistically significant and are shown in bold.

**Supplementary Table 4.** Correlation between each gene in SLE. The correlation coefficients in SLE (n=19) are shown between two genes, and ρ-values were determined by nonparametric Spearman’s rank correlation test. P values <0.05 were considered statistically significant and are shown in bold.

**Supplementary Table 5.** Correlation between each gene in DM. The correlation coefficients in DM (n=24) are shown between two genes, and ρ-values were determined by nonparametric Spearman’s rank correlation test. P values <0.05 were considered statistically significant and are shown in bold.

**Supplementary Table 6. Clinical characteristics of the enrolled patients with DM.**


A flare was defined as an elevation of creatine kinase value with muscle weakness and/or muscle pain as a myositis or exacerbation of ILD. DM: dermatomyositis, CADM: clinically amyopathic dermatomyositis, ARS: aminoacyl-tRNA synthetase, MDA5: melanoma differentiation-associated gene 5, TIF1γ: transcriptional intermediary Factor 1 γ, PSL: prednisolone, Aza: azathioprine, Tac: tacrolimus, IVCY: intravenous cyclophosphamide, ILD: interstitial lung disease, -: negative, ND: not defined.

**Supplementary Table 7. Clinical characteristics of the enrolled patients with SLE.**

Anti-Sm antibodies (normal index value (-) <7 ; indeterminate (±): ≤7 to <29; positive (+): ≥30), anti-RNP antibodies (-<15 ; ±: ≤15 to <21; +: ≥22) and anti-SSA/Ro antibodies (- <10 ; ±: ≤10 to <29; +: ≥30) were measured with a chemiluminescent enzyme immunoassay. A flare was defined as an increase of 3 or more points of SLEDAI compared to the last visit. SLE: systemic lupus erythematosus, ANA: antinuclear antibody, SP: speckled pattern, H: homogeneous, HSP: homogeneous and speckled pattern, RNP: ribonucleoprotein, SS-A: Sjögren's syndrome-related antigen A, dsDNA: double-stranded DNA, CNS: central nervous system, SLEDAI: SLE Disease Activity Index, PSL: prednisolone, mPSL-P: methylprednisolone pulse, -: negative, ND: not defined.

**Supplementary Table S8. Clinical characteristics of the enrolled patients with AIBD.**

A flare was defined as a flare-up of any clinical symptoms with the elevation level of the specific antibody, such as vesicles and/or multiple edematous erythema. AIBD: autoimmune blistering disease, PV: pemphigus vulgaris, BP: bullous pemphigoid, PV: pemphigus foliaceus, Dsg: desmoglein, PSL: prednisolone, PE: plasma exchange, IVIG: intravenous immune globulin, AZA: azathioprine, mPSL-P: methylprednisolone pulse, -: negative, ND: not defined.

**Supplementary Table S9.** Primers used in this study.
